# Differences in Sepsis Treatment and Outcomes between Public and Private Hospitals in Brazil: A Multicenter Observational Study

**DOI:** 10.1371/journal.pone.0064790

**Published:** 2013-06-06

**Authors:** Katia Aparecida Pessoa Conde, Eliezer Silva, Carla Oliveira Silva, Elaine Ferreira, Flavio Geraldo Rezende Freitas, Isac Castro, Alvaro Rea-Neto, Cintia Magalhaes Carvalho Grion, Anselmo Dornas Moura, Suzana Margareth Lobo, Luciano Cesar Pontes Azevedo, Flavia Ribeiro Machado

**Affiliations:** 1 Departamento de Anestesiologia, Dor e Terapia Intensiva. Universidade Federal de São Paulo, São Paulo, SP, Brazil; 2 Latin-America Sepsis Institute, São Paulo, SP, Brazil; 3 Centro de Estudos e Pesquisas em Terapia Intensiva (CEPETI), Curitiba, PR, Brazil; 4 Hospital Universitário de Londrina, Divisão de Terapia Intensiva, Londrina, PR, Brazil; 5 Intensive Care Unit, Hospital Mater Dei, Belo Horizonte, MG, Brazil; 6 Serviço de Terapia Intensiva, Faculdade de Medicina de São José do Rio Preto, Hospital de Base, São Jose do Rio Preto, SP, Brazil; D'or Institute of Research and Education, Brazil

## Abstract

**Background:**

Previous studies showed higher sepsis mortality rates in Brazil compared to other developed or developing countries. Moreover, another trial demonstrated an increased mortality rate in public hospitals compared to private hospitals in Brazil. The reasons for these findings may include delayed recognition and inadequate treatment of sepsis in public facilities. We designed this study to evaluate the factors associated with mortality in septic patients admitted to intensive care units in a network of public and private institutions.

**Materials and Methods:**

This study is a retrospective analysis of a prospective cohort of sepsis patients in 19 private and public institutions in Brazil. We analyzed data from the original database and collected additional data to assess compliance to the treatment guidelines and to determine the time from the onset of organ dysfunction and the sepsis diagnosis by the healthcare team.

**Results:**

A total of 396 patients were analyzed. Patients in public hospitals were younger, had a greater number of dysfunctional organs at baseline and a lower chance to have sepsis diagnosed within two hours of the onset of organ dysfunction. Private hospitals had a better compliance to lactate and blood culture sampling and maintenance of glycemic control. The multivariate analysis showed that age, disease severity at baseline and being treated at a public hospital were independent risk factors for mortality. A delay in the sepsis diagnosis of longer than two hours was associated with mortality only in the public setting.

**Conclusions:**

We confirmed a lower sepsis mortality rate in the private hospitals of this network. Being treated in a public hospital was an independent factor for mortality. Delayed recognition of sepsis was more frequent in public institutions and this might have been associated with a higher mortality. Improving sepsis recognition and early diagnosis may be important targets in public institutions.

## Introduction

Sepsis is the leading cause of death in non-cardiac intensive care units (ICUs) around the world, with high death rates especially in underdeveloped and developing countries [1]–[5]. In Brazil, several studies already showed high mortality rates [6]–[8] that are even higher than in other developing countries such as Argentina and India [9]. The reasons for this finding are not clear. A case mix can partially explain this observation in Brazil because there are significant inequalities in access to health care and a shortage of ICU beds [10]. In addition, all previous epidemiological studies included only ICU patients, which are possibly the most severe cases of sepsis. However, this high mortality could also be associated with delayed recognition and inadequate treatment of sepsis.

In a previous multicenter study, a difference in mortality rates between the public and private hospitals in Brazil was clearly demonstrated [6]. However, only five centers were involved, and from these, only one was a private institution. In the COSTS study, an epidemiological cohort trial aiming to analyze sepsis-related costs in 21 Brazilian institutions, the mortality rates for the public and private hospitals were significantly different (49.1% and 36.7%, respectively) [7]. Possible reasons for these findings are differences in the profiles of the two healthcare systems, differences in the treated populations, promptness of the diagnosis or adequacy of treatment. Although differences in mortality rates in teaching and non-teaching hospitals were shown [1], [11], differences related to the type of healthcare system (public vs. private) were not clearly reported. Thus, this study aimed to identify the risks factors associated with sepsis mortality in these two different types of institutions in Brazil using the COSTS database, a robust multicenter observational prospective study.

## Methods

This study is a post-hoc analysis of the patients included in the COSTS study, which was carried out in 21 Brazilian public and private ICUs [7]. The original trial screened all of the patients admitted to these ICUs from October 1, 2003 to March 30, 2004. All patients older than 18 years of age with a diagnosis of sepsis, severe sepsis or septic shock were prospectively included after signed informed consent. Briefly, patients were eligible for inclusion if they had two or more signs of systemic inflammatory response syndrome secondary to a known or suspected infection. The presence of at least one sepsis-induced organ dysfunction was required for the diagnosis of severe sepsis and fluid refractory hypotension and vasopressors use characterized the diagnosis of septic shock. Patients were excluded if they were pregnant or if their physicians were not committed to full life support. In this posthoc analysis, we analyzed only the severe sepsis and septic shock patients.

Sepsis-induced organ dysfunction, assessed at the moment of sepsis diagnosis, was considered to be one of the following: hypotension, an arterial oxygen partial pressure/oxygen inspiratory fraction (PaO_2_/FiO_2_) ratio ≤300, lactate level ≥1.5 times the reference value and base deficit >5, bilirubin level >2 times the reference value, urine output ≤0.5 ml/kg/hour after adequate volume replacement or the need for renal replacement therapy, platelet count ≤100,000 mm^3^ or a decrease of 50% from the last 3 days’ values, or a reduced level of consciousness. Septic shock was defined as volume-refractory hypotension with need for vasopressors at any time during the sepsis episode. We considered public institutions to be hospitals in which the main source of revenue was the National Healthcare System (Sistema Unico de Saude – SUS) and private institutions to be hospitals in which the main billing system was based on health insurance.

Information related to the time to sepsis diagnosis and adequacy of treatment were retrospectively obtained from medical records. The time to the sepsis diagnosis was defined as the number of hours elapsed between the onset of the first organ dysfunction and the recognition and management of sepsis by the healthcare provider. We defined sepsis recognition as the documentation of a sepsis diagnosis in the patient’s chart. To identify the timing of organ dysfunction, the charts were carefully reviewed to determine the first recording of hypotension, reduced level of consciousness or low urine output as well as the first laboratory sampling time in which the results fulfilled the respiratory, metabolic, coagulation or hepatic criteria for organ dysfunction. In patients admitted from the emergency department already with severe sepsis criteria, we used the time of triage. The SOFA scores [12]were determined at the beginning of organ dysfunction and at the time of the sepsis diagnosis. We determined the delta SOFA as the difference between the SOFA at the onset of dysfunction and the SOFA at the time of the sepsis diagnosis.

We also assessed the compliance with the Surviving Sepsis Campaign (SSC) [13] bundles, considering the moment of the sepsis diagnosis as the baseline. We used the SSC criteria to define adherence [14]. Time to antibiotics was calculated as the time elapsed between the diagnosis of severe sepsis/septic shock and the first dose of intravenous antibiotics. This analysis was restricted to those patients not under antibiotics or those who had previous antibiotics changed because of the current sepsis episode. In the 24–h bundles, only the compliance to glycemic control was analyzed. We did not evaluate the compliance to corticosteroids and activated protein C as, according to SSC, this is defined by following or not institutional protocols. However, we collected data on their use. The data for plateau pressure was inconsistent in the charts; thus, this parameter was not collected.

To minimize possible biases in the retrospective data collection, especially regarding the determination of the onset of organ dysfunction and the time of the sepsis diagnosis, data were collected by only two authors (KC for 89% and FGRF for 11% of the sites). They were not blind to the patients’ identification and mortality outcome, as this information was available in the charts. However, they were instructed to check the information on the sepsis episode before assessing mortality. Although they collected data on their own hospitals, they were not in charge of those patients during the original study. Any controversy was discussed with the senior author (FRM).

All other data were retrieved from the original database, including the costs analysis. In the original study, we assessed all enrolled patients daily, analyzing cost related expenditures and all interventions were priced in a standardized fashion. This strategy allowed us to use costs as a measurement of the intensity of clinical support and quality of care, thus we included it in our risk factor analysis.

For the present study, all of the original sites were invited to participate. The Research Ethics Committee from the coordinating institution (Comitê de Ética em Pesquisa - UNIFESP) and all of the other institutions that agreed to participate in the present study approved the protocol and waived the need for a new informed consent.

### Statistical Analysis

We described categorical variables as percentages and continuous variables by their central tendency and dispersion according to their distribution, as assessed by the Kolmogorov-Smirnov test. We used the Mann-Whitney and Pearson chi squared tests for continuous and categorical data, as appropriate.

Our aim was to address the differences between public and private institutions, as well as the risk factors for mortality. Thus, we did two different multivariate logistic stepwise forward regression models. In the first one, we assess the characteristics that were independently associated with each type of institution. In the second, we analyze the risk factors for mortality in the global population as well as in each type of institution. All of the variables in the univariate analysis that had a p value below 0.2 were included in a multivariate logistic stepwise forward regression model. In this analysis, we decided not to include variables with missing data for more than 50 patients because the lack of data would result in serious inconsistencies. The model calibration was assessed using the Hosner-Lemeshow test, which was considered to be appropriate if p above 0.10. The time to the sepsis diagnosis was categorized using the receiver operating characteristic (ROC) curve for mortality. Because having sepsis diagnosed within either one or two hours had a similar sensitivity and specificity, we run separate multivariate analyses using both variables as well as the original continuous values. We chose to express the results with the two-hour cut off in the comparison between the public and private hospitals because these results had a better calibration in the Hosner-Lemeshow test. However, we also reported the OR (IC 95%) results for the 1-hour cut off when this variable remained in the model. We did a collinearity diagnosis and also run the model with/without hospital type to assess the changes in odds ratios among the other variables.

The patient clustering in two groups (public and private hospital) resulted in two imbalanced groups (Table 1). In order to adjust for these differences, we fit a propensity score [**15**], [16]. Propensity score analyses are able to take into account as much variables related to the outcome as needed, reducing bias [17]. We included variables related both to patient location (public or private) and to outcome (hospital mortality). To assess the balancing of covariates between public and private groups in each propensity score quintile, we drew side-by-side box plots of the estimated propensity scores for patients from public and private hospitals within each quintile of the propensity scores [16]. To generate the propensity score we included in a multivariate logistic regression all variables related both to the exposure and the outcome with P value less than 0.20 in the univariate analysis in both table 1 and 2 [16], [18]. We decide to include variables related to outcome as this would possible add to our score variables linked to some other relevant and unknown variables related to exposure. This inclusion would increase the precision of the estimated exposure effect without increasing bias according to recent findings. [16], [19] The score was finally entered as a continuous variable into the logistic regression model, with hospital mortality as dependent variable and patient location as independent variable [15], [16], [20]. This logistic regression analysis took into account the hospital clustering effect. We evaluated the calibration ability for the model using the Hosmer-Lemeshow goodness-of-fit test.

**Table 1:** Main characteristics according to the type of hospital.

Variable	Patients at public hospitals (N = 258)	Patients atprivate hospitals (N = 138)	Univariate analysisp value	Multivariate analysisp value OR(CI95%)
Age (years)	59 (44–73)	68 (51–81)	0.0004	<0.00011.03 (1.01–1.04)
Male gender	159 (61.6)	80 (58.0)	0.54	–
APACHE II	23 (21–28)	21 (16–27)	0.15	NS
SOFA dysfunction	6 (5–9)	6 (4–8)	0.22	–
SOFA diagnosis	7 (5–10)	7 (4–9)	0.04	NS
DeltaSOFA	0.0(0.0–1.0)	0.0(0.0–0.0)	0.02	NS
Comorbidities	194 (75.2)	85 (85.9)	0.04	NA
Respiratory	26 (10.1)	9 (9.1)	0.93	–
Renal	30 (11.8)	15 (15.3)	0.48	–
Cardiovascular	91 (30.5)	50 (50.5)	0.01	NA
Neurological	26 (10.4)	22 (22.2)	0.006	NA
Immunossuppression	66(25.6)	23 (23.2)	0.74	–
Diabetes mellitus	44 (17.3)	18 (18.2)	0.97	–
Hepatic	19 (7.4)	6 (6.1)	0.84	–
Classification				
Clinical	142 (54.9)	62 (62.6)	0.22	–
Surgical	116 (45.1)	37 (37.4)		
Septic shock	118 (45.7)	81 (58.7)	0.01	NS
Infection type				
Community-acquired	112 (43.4)	69 (50.0)	0.22	NS
Nosocomial (ward)	103 (39.9)	43 (31.2)		
Nosocomial (ICU)	43 (16.7)	26 (18.8)		
Site of diagnosis				
Emergency room	73 (28.3)	48 (34.8)	0.36	–
Ward	118 (45.7)	60 (43.5)		
ICU	67 (26.0)	30 (21.7)		
Source of infection				
Pulmonary	134 (51.9)	70 (50.7)	0.82	–
Intraabdominal	64 (24.8)	14 (10.1)	0.001	0.0200.40 (0.19–0.87)
Urinary	20 (7.8)	18 (13.0)	0.11	NS
Other	40(15.5)	36 (26.2)		
Organ dysfunction (n)	2.0(2.0–3.0)	2.0(1.0–3.0)	<0.0001	0.0050.70 (0.55–0.89)
Cardiovascular	148 (57.4)	76 (55.1)	0.73	–
Respiratory	212 (82.2)	105 (76.1)	0.18	NS
Renal	114 (44.2)	43 (31.2)	0.01	NS
Metabolic	68 (26.4)	22 (15.9)	0.02	NS
Hematological	72 (27.9)	25 (18.1)	0.04	NS
Hepatic	50 (19.4)	10 (7.2)	0.002	NS
Time to the sepsis diagnosis (h)	6(1.2–20.0)	3(0.0–11.0)	0.01	NA
Diagnosis in one hour	61 (23.9)	54 (39.1)	0.002	0.0321.85 (1.05–3.33)
Diagnosis in two hours	71(27.8)	61 (44.2)	0.001	0.0491.75(1.01–3.03)
Compliance with the sepsis bundles*				
Lactate sampling	39 (15.1)	77 (55.8)	<0.0001	<0.00015.59(3.16–9.91)
Blood cultures	42 (16.3)	47 (34.1)	<0.0001	<0.00013.26(1.75–6.09)
Antibiotics	164 (63.6)	61 (44.6)	<0.0001	NS
Time from diagnosis to antibiotics (h)**	0 (0–4)	2.3 (0–8)	<0.0001	NA
Time from dysfunction to antibiotics (h)**	10.8 (3.5–29.3)	8,5(3.0–19.9)	p = 0.267	NA
Fluid resuscitation	107 (71.3)	56 (60.2)	0.09	NA
Vasopressors	94 (81.0)	65 (79.3)	0.89	–
CVP optimization	15 (13.0)	31 (37.3)	<0.0001	NA
ScvO_2_ optimization	2 (1.8)	12 (14.6)	0.001	NA
Glycemic control	67 (26.0)	72 (52.2)	<0.0001	0.0012.45(1.42–4.24)
Corticosteroids use	26(23.4%)	27(34.2%)	0.14	NA
Drotrecogin use	5(1.9%)	2(1.5%)	0.73	–
Entire 6-h bundle	1 (0.4)	2 (1.4)	0.58	–
Daily costs (R$)***	1,835.9(1,478.1–2,273.4)	2,041.2(1,504.6–2,781.7)	0.003	0.0021.01(1.00–1.01)
Total costs (R$)***	19,922.2(9,255.2–41,118.2)	17,335.0(9,186.5–35,480.9)	0.32	–
Length of ICU stay				
ICU mortality				
Hospital mortality	157 (60.9)	65 (47.1)	0.01	0.0050.44 (0.25–0.78)

APACHE–Acute Physiological and Chronic Health Evaluation, SOFA–Sequential Organ Failure Assessment, ICU–intensive care unit, CVP–central venous pressure, ScvO–central venous oxygen saturation, OR–odds ratio, CI–confidence interval, NS–non-significant, NA–not applicable, significant variable in the univariate analysis but not included in the multivariate analysis due to missing data. The results are expressed as a number (%) or median (25%–75%). *Total number of patient variables according to the bundle evaluated [(fluid resuscitation (n = 243), vasopressors (n = 198), CVP and Scv0? (n = 197)]. ** only those not in previous antibiotics use or in whom the previous antibiotics was changed. (public: n = 115, public: n = 187).*** Approximately currency: 1R = 1.8 US. Chi-squared and Mann Whitney tests (univariate). Diagnosis within one hour was included in the multivariate analysis. Multivariate analysis with stepwise forward regression, reference: private hospitals. Hosmer-Lemeshow test p = 0.41.

**Table 2:** Risk factors for hospital mortality (global analysis).

Variable	Survivors(N = 174)	Non-survivors(N = 222)	Univariate analysisp value	Multivariate analysisp value–OR(CI95%)
Age (years)	57 (39–70)	66 (51–70)	<0.0001	<0.00011.03 (1.02–1.04)
Male gender	102 (58.6)	137 (61.7)	0.53	–
APACHE II	19 (14–23)	24 (19–29)	<0.0001	0.0021.05 (1.02–1.08)
SOFA dysfunction	6 (4–8)	7 (5–9)	<0.0001	NS
SOFA diagnosis	6 (4–8)	8 (6–10)	<0.0001	NS
Comorbidities	101 (67.3)	178 (86.0)	<0.0001	NA
Respiratory	8 (5.3)	27 (13.0)	0.01	NA
Renal	13 (8.7)	32 (15.8)	0.04	NA
Cardiovascular	56 (37.3)	85 (41.1)	0.47	–
Neurological	27 (18.0)	21 (10.6)	0.04	NA
Immunossuppression	31 (20.7)	58 (28.0)	0.11	NA
Diabetes mellitus	25 (16.7)	37 (18.2)	0.70	–
Hepatic	6 (4.0)	19 (9.3)	0.05	NA
Classification				
Clinical	77 (51.3)	126 (61.2)	0.08	NA
Surgical	73 (48.7)	80 (38.8)		
Septic shock	86 (49.4)	113 (50.9)	0.77	–
Infection type				
Community-acquired	89 (51.2)	92 (41.5)	0.07^#^	NS
Nosocomial (ward)	54 (31.0)	92 (41.5 )		
Nosocomial (ICU)	31 (17.8)	38 (17.0)		
Site of diagnosis				
Emergency Room	60 (34.5)	61 (27.5)	0.25	–
Ward	71 (40.8)	107 (48.2)		
ICU	43 (24.7)	54 (24.3)		
Source of infection				
Pulmonary	84 (48.3)	120 (54.1)	0.66	–
Intraabdominal	35 (20.1)	43 (19.1)		
Urinary	19(10.9)	19 (8.6)		
Other	47 (20.7)	48 (18.2)		
Organ dysfunction	2.07±1.1	2.63±1.2	<0.0001	NS
Cardiovascular	90 (51.7)	134 (60.4)	0.08	NS
Respiratory	136 (78.2)	181 (81.5)	0.40	–
Renal	54 (31.0)	103 (46.4)	0.002	NS
Metabolic	35 (20.1)	55 (24.8)	0.27	–
Hematological	28 (16.1)	69 (31.1)	<0.0001	0.0241.93(1.09–3,41)
Hepatic	18 (10.3)	42 (18.9)	0.01	NS
Hospital characteristics				
Public	101(58.0)	157(70.7)	0.009	0.0012.55 (1.50–4.32)
Private	73 (42.0)	65 (29.3)	0.034	
Daily costs(R$)***	1,696.0(1,377.0–2,003,7)	2,070.9(1,644,2–2,599,9)	0.0001	<0.00011.001(1.001–1.001
Total costs(R$)***	20,578,0(7,966,8–34,063,7)	19,085(10,092,0–41,872,1)	0.22	–
Time to the sepsis diagnosis (h)	4 (0–12)	6 (11–21)	0.006	NS
Diagnosis in one hour	62 (35.8)	53 (24.1)	0.01	NS
Diagnosis in two hours	68(39.3)	64(29.1),	0.03	NS
Compliance with the sepsis bundles*				
Lactate sampling	57 (32.8)	59 (26.6)	0.18	NS
Blood cultures	38 (21.8)	51 (23.0)	0.78	–
Antibiotics	99 (56.9)	126 (56.8)	0.97	–
Time from diagnosis to antibiotics (h)**	0.48 (0–4.56)	0 (0–6.48)	0.36	–
Fluid resuscitation	75 (72.1)	88 (63.3)	0.15	NA
Vasopressors	67 (80.7)	92 (80.0)	0.95	–
CVP optimization	26 (30.6)	20 (17.7)	0.05	NA
ScvO_2_ optimization	7 (8.3)	7 (6.2)	0.76	–
Glycemic control	74 (42.5)	65 (29.3)	0.006	0.0260.57 (0.35–0.93)
Entire 6-h bundle	2 (1.1)	1 (0.5)	0.83	–

APACHE–Acute Physiological and Chronic Health Evaluation, SOFA–Sequential Organ Failure Assessment, ICU–intensive care unit, CVP–central venous pressure, ScvO–central venous oxygen saturation, OR–odds ratio, CI–confidence interval, NS–non-significant, NA–not applicable, significant variable in the univariate analysis but not included in the multivariate analysis due to missing data. The results are expressed as a number (%) or median (25%–75%). *Total number of patient variables according to the bundle evaluated [(fluid resuscitation (n = 243), vasopressors (n = 198), CVP and Scv0? (n = 197)]. ** only those not in previous antibiotics use or in whom the previous antibiotics was changed. (public: n = 115, public: n = 187).*** Approximately currency: 1R = 1.8 US. Chi-squared and Mann Whitney tests (univariate). Multivariate analysis with stepwise forward regression, reference: non-survivors. Hosmer-Lemeshow test p = 0.19.

Statistical analysis was done using SPSS 17.0 package for Windows and GraphPad Prism 5 for Windows ® - version 5.0–2007.

## Results

**Figure 1:**
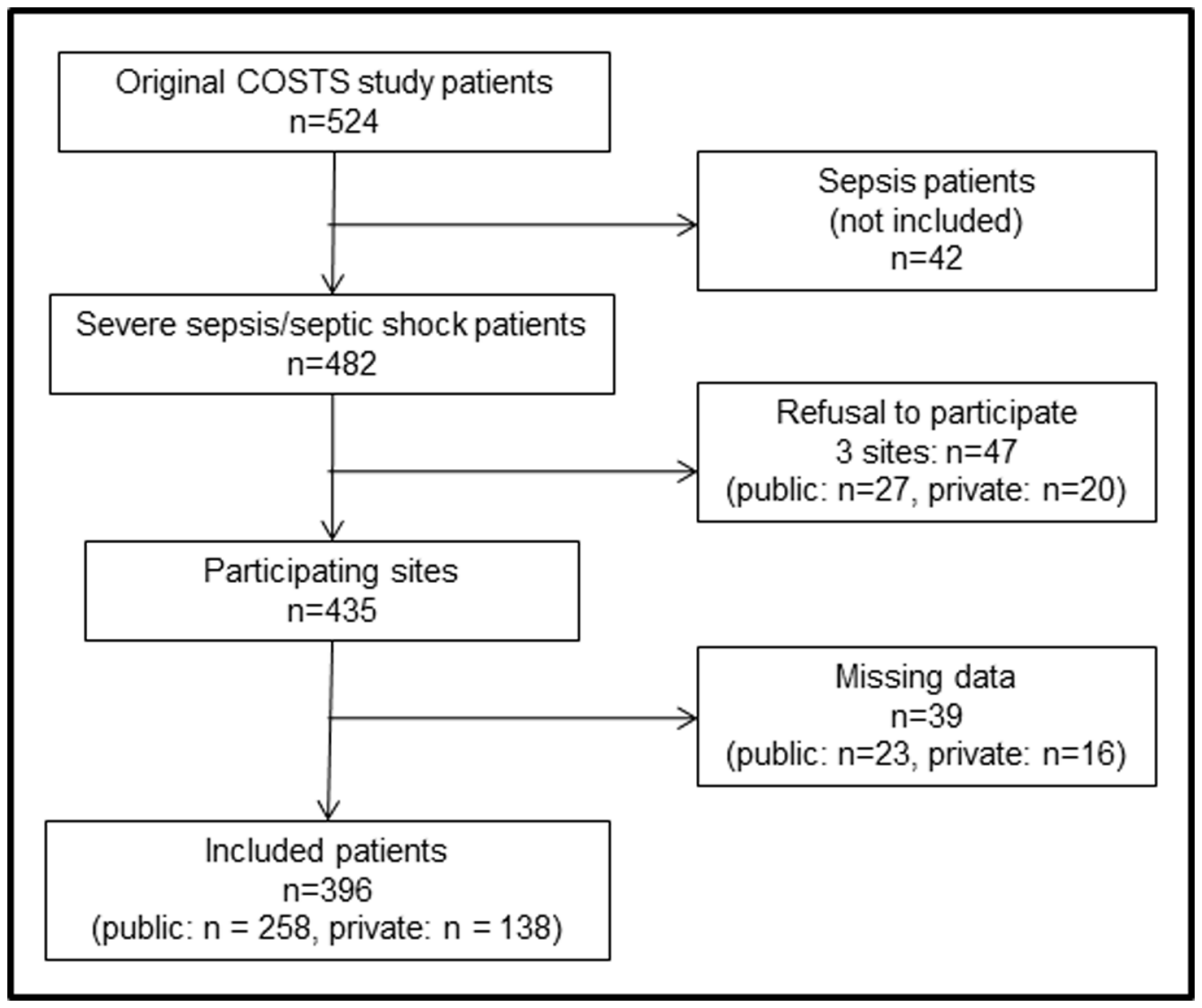
Study flowchart.

We analyzed 396 patients out of 524 who were originally included in the COSTS study. Forty-two patients were not included because they had sepsis without organ dysfunction. Three of the original centers did not agree to participate in this study, two public and one private, totaling 47 patients. In addition, data were missing for 39 patients from the current participating sites. To summarize, a total of 86 patients with severe sepsis or septic shock were not included in the present study (Figure 1). Ultimately, we included patients from 18 of the 21 original sites. Of these sites, nine centers were characterized as public hospitals and nine as private hospitals. The participating centers are listed in Table S1.

### Differences between the Public and Private Hospitals

According to the multivariate analysis, there were some characteristics significantly associated with the public and private hospitals (Table 1). Patients admitted to private hospitals were older and were more likely to be diagnosed with sepsis within two-hours of the onset of organ dysfunction. In addition, private hospitals had higher daily costs and a better compliance to lactate and blood culture sampling and maintenance of glycemic control. In contrast, public hospitals had a higher prevalence of intraabdominal infections, more organs with dysfunction and higher mortality. In the univariate analysis, the SOFA score at the onset of organ dysfunction was similar between the two types of hospitals (Table 1). However, the SOFA score, delta SOFA and the number of organs with dysfunction at the moment of diagnosis were higher in the public hospitals.

### Assessment of Mortality Risk Factors

In the multivariate analysis, older age, higher APACHE II score, higher daily costs, poorer glycemic control, the presence of hematological dysfunction and being admitted to a public hospital were associated with a higher mortality (Table 2). The time to the sepsis diagnosis did not remain in the multivariate model. There was no relevant collinearity between the variables included in the model.

**Figure 2:**
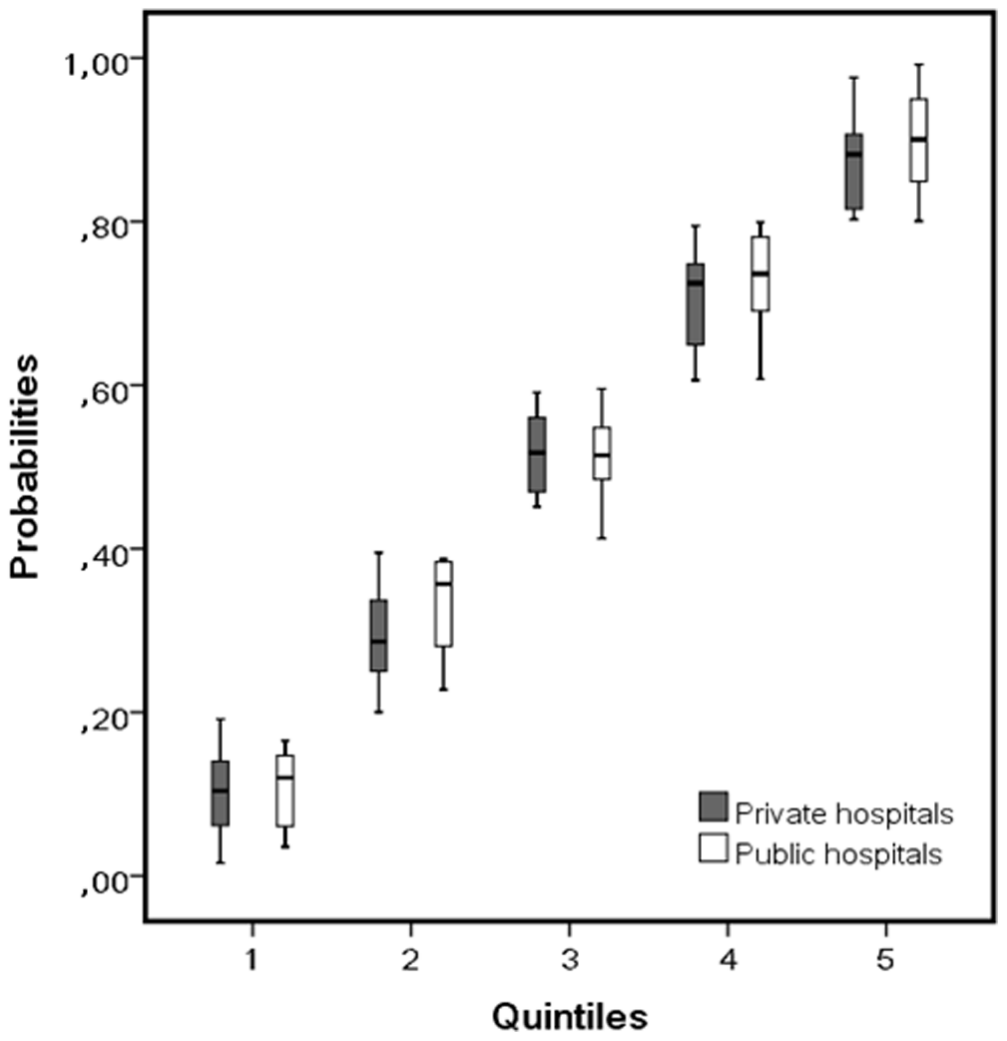
Comparison of propensity scores for patients from public and private hospitals within each propensity score quintile. The groups are comparable because there is sufficient overlap in the propensity score within each block.

The propensity score was as patients and their scores were equality distributed among the score quintiles within the groups (Figure 2). Our model has an AUC of 0.846 to predict the type of hospital. The logistic regression analysis with hospital mortality as dependent variable and patient location as independent variable, including the propensity score as a continuous variable confirmed the type of institution as a independent factor associated with mortality (OR 1.719 CI95%: 1.040–2.843, p = 0.036), with a good calibration by the Hosmer-Lemeshow test (p = 0.841). We also separately analyzed the factors associated with mortality in each type of hospital (Table 3). In the public hospitals, in the multivariate analysis, the variables associated with mortality were age, APACHE II score, the presence of hematologic dysfunction and daily costs. Compared to the non-survivors, among the survivors, there was a higher proportion of patients in whom the sepsis diagnosis was made either within one or two hours. In the private hospitals, the variables that remained in the logistic regression model were only age, SOFA score at the time of diagnosis, daily costs and glycemic control. As opposite of the findings in public hospitals, the time to the sepsis diagnosis was not different between the survivors and non-survivors in the private setting (Table 3).

**Table 3:** Risk factors for hospital mortality according to the type of institution.

	Public hospitals			Private hospitals		
Characteristics	Survivors(n = 101)	Non-survivors (n = 157)	p valueOR (IC 95%)**	Survivors(n = 73)	Non-survivors (n = 65)	p valueOR (IC 95%)**
Age (years)	52 (38–66)	62 (50–76)	0.0011.03(1.01–1.04)	62 (47–76)	72 (58–86)	0.0011.04(1.02–1.06)
Male gender	63 (62.4)	96 (61.1)		39 (53.4)	41 (63.1)	–
APACHE II	19,0 (14,0–24,0)	25,0 (20,0–30,0)	0.0021.07(1.02–1.11)	19.0 (14.0–25.0)	23.0 (18.0–28.0)	NS
SOFA dysfunction	6.0 (4.0–8.0)	7.0 (5.0–9.0)	NS	5.0 (3.0–8.0)	7.0 (5.0–10.0)	NS
SOFA diagnosis	6.0 (5.0–9.0)	8.0 (6.0–10.0)	NS	5.0 (3.0–8.0)	8.0 (6.0–10.0)	0.0031.221(1.07–1.40)
Comorbidities	63 (62.4)	131 (83.4)	NA	38 (77.6)	47 (94.0)	NA
Respiratory	6 (5.9)	20 (12.7)	–	2 (4.1)	7 (14.0)	–
Renal	6 (5.9)	24 (15.7)	NA	7 (14.6)	8 (16.0)	–
Cardiovascular	31 (30.7)	60 (38.2)	–	25 (51.0)	25 (50.0)	–
Neurological	14 (13.9)	12 (19.0)	–	13 (26.5)	9 (18.0)	–
Immunossuppression	19 (18.8)	47 (29.9)	NA	12 (24.5)	11 (22.0)	–
Diabetes mellitus	15 (14.9)	29 (8.1)	NA	10 (20.4)	8 (16.0)	–
Hepatic	6 (5.9)	13 (8.4)	–	0 (0.0)	6 (12.0)	NA
Classification						
Clinical	49 (48.5)	92 (59.0)	NA	28 (57.1)	34 (68.0)	–
Surgical	52 (51.5)	64 (41.0)		21 (42.9)	16 (32.0)	–
Septic shock	44 (43.6)	74 (47.1)		42 (57.5)	39 (60.0)	–
Infection type						
Community–acquired	50 (49.5)	62 (39.5)	NS	39 (53.4)	30 (46.2)	–
Nosocomial (ward)	30 (29.7)	73 (46.5)		24 (32.9)	19 (29.2)	–
Nosocomial (ICU)	21 (20.8)	22 (14.0)		10 (13.7)	16 (24.6	–
Site of diagnosis						
Emergency room	32 (37.1)	41 (26.1)	–	28 (38.4)	20 (30.8)	–
Ward	40 (39.6)	78 (49.7)		31 (42.5)	29 (44.6)	–
ICU	29 (28.7)	38 (24.2)		14 (19.2)	16 (24.6)	–
Source of infection						
Pulmonary	50 (49.5)	84 (53.5)	–	34 (46.6)	34 (55.4)	–
Intraabdominal	26 (25.7)	39 (24.8)		9 (12.3)	5 (7.7)	
Urinary	9 (8.9)	11 (7.0)		10 (13.7)	8 (12.3)	
Other	16 (15.8)	24 (15.3)		20 (27.4)	16 (24.6)	
Organ dysfunction	2.22±1.2	2.80±1.2	NS	1.87±1.0	2.21±0.9	NS
Cardiovascular	53 (52.5)	95 (60.5)	NS	37 (50.7)	39 (60.0)	–
Respiratory	80 (79.2)	132 (84.1)	–	56 (76.7)	49 (75.4)	–
Renal	34 (33.7)	80 (51.0)	NS	20 (27.4)	23 (35.4)	–
Metabolic	24 (23.8)	44 (28.0)	–	11 (15.1)	11 (16.9)	–
Hematological	17 (26.8)	55 (35.0)	0.0412.08(1.03–4.20)	11 (15.1)	14 (21.5)	–
Hepatic	16 (15.8)	34 (21.7)	–	2 (2.7)	8 (12.3)	NS
Daily costs(R$)***	1,649.9(1,319,7–1,895.4)	1,978.3(1,612.7–2.395.0)	0,0011.001(1.000–1.001)	1,781.3(1,463.3–2,198.9)	2,412.2(1,894.2–3,035.4)	<0.00011.001(1.000–1.002)
Total costs(R$)***	21,818.3(8,441.0–39,593.3)	19,055.4(10.032,9–41.118,2)	–	16,518.6(7,569.6–28,129.3)	19,267.6(10,236.6–42,071.1)	–
Time to the sepsis diagnosis (h)	5(0.48–13.00)	8(2.32–29.00)	NS	3.36 (0.0–10.24)	2.32 (0.0–11.28)	–
Diagnosis in one hour	35 (35)	26 (16.8)	0.0040.37 (0.19–0.73)	27 (37.0)	27 (41.5)	–
Diagnosis in two hours	39(39.0)	32(20.6)	0.0050.40(0.22–0.77)	29(39.7)	32(49.2)	–
Compliance with the sepsis bundles*						
Lactate sampling	15 (14.9)	24 (15.3)	–	42 (57.5)	35 (53.8)	–
Blood cultures	13 (12.9)	29 (18.5)	–	25 (34.2)	22 (33.8)	–
Antibiotics	67 (66.3)	97 (61.8)	–	32 (43.8)	29 (44.6)	–
Time from diagnosis to antibiotics (h)**	0 (0–2.40)	0 (0–4.08)	–	2.16 (0–5.52)	2.16 (0–10.32)	–
Fluid resuscitation	42 (79.2)	65 (67.0)	NA	33 (64.7)	23 (54.8)	–
Vasopressors	33 (80.5)	61 (81.3)	–	34 (81.0)	31 (77.5)	–
CVP optimization	6 (14.6)	9 (12.2)	–	18 (42.9)	11 (28.2)	–
ScvO_2_ optimization	1 (2.4)	1 (1.4)	–	6 (14.0)	6 (15.4)	–
Glycemic control	27 (26.7)	40 (25.5)	–	47 (64.4)	25 (38.5)	<0.00010.20(0.08–0.49)
Entire 6-h bundle	0(0)	1 (0.6)	–	2 (2.4)	0 (0)	–

APACHE–Acute Physiological and Chronic Health Evaluation, SOFA–Sequential Organ Failure Assessment, ICU–intensive care unit, CVP–central venous pressure, ScvO–central venous oxygen saturation, OR–odds ratio, CI–confidence interval, NS–non-significant, NA–not applicable, significant variable in the univariate analysis but not included in the multivariate analysis due to missing data. The results are expressed as a number (%) or median (25%–75%).* Total number of patients variable according to the bundle. **Multivariate analysis with stepwise forward regression, reference: non-survivors. ** only those not in previous antibiotics use or in whom the previous antibiotics was changed. (public: n = 115, public: n = 187).*** Approximately currency: 1R = 1.8 US. Hosmer-Lemeshow for public hospitals p = 0.93, private hospitals p = 0.26.

**Figure 3:**
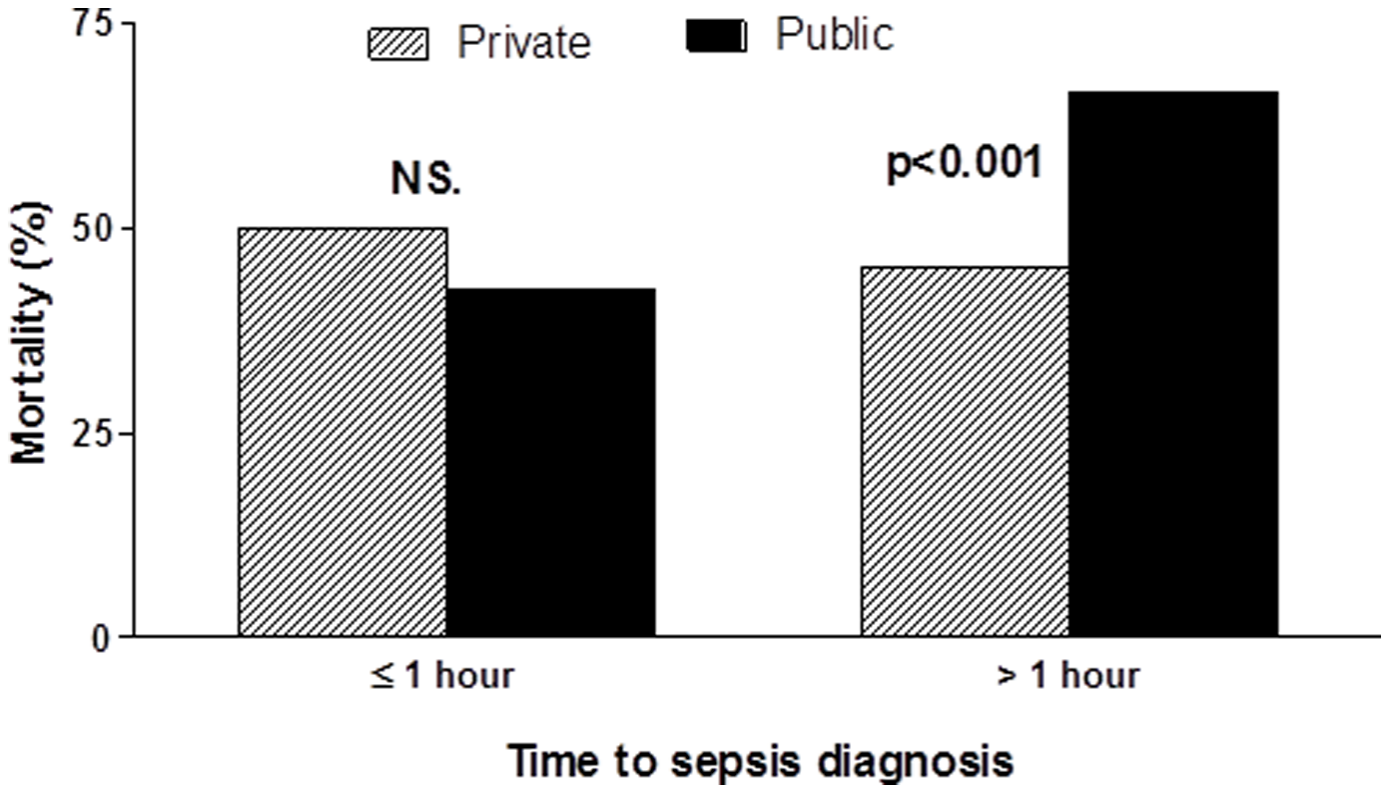
Mortality according to the time of the sepsis diagnosis in public and private hospitals. NS: Non-significant.

Although the global mortality rates were higher in the public hospitals, there was no difference when we evaluated the patients in whom the diagnosis of sepsis was made within one hour of the onset of organ dysfunction (42.6% vs. 50% for public and private institutions, p = 0.45). Thus, this significant difference between the two types of hospitals is solely due to increased mortality in the public setting among those diagnosed after one hour (66.5% vs. 45.2%, p<0.001) (Figure 3). These results remained unchanged when the two-hour cut-off was used (data not shown).

As we missed data for 86 patients in the retrospective analysis, we also compared those patients with our main group (n = 396). There were no significant differences between the groups in age, gender, APACHE II, SOFA score or the distribution according to the type of hospital (public or private). There were 18 patients still alive at the ICU on day 60 (main group: 16(4.0%); missing group: 2(2.3%, p = 0.414). In the mortality assessment, we adopted a conservative approach and considered dead all patients still in the ICU at the 60th day in the subgroup of missing patients. In the main group, as we have data on hospital mortality, we considered all patients that died in the hospital as died during ICU stay. There was no significant difference in mortality rates between the two groups (main group: 49.5%, missing group: 44.2%, p = 0.371). The difference in mortality rates between the two types of institutions remained significant when we analyzed the whole group (n = 482: public hospitals: 52.4% vs. private hospitals: 41.6%, p = 0.023) (Table S2).

## Discussion

In this study, septic patients admitted to private hospitals in Brazil had lower mortality rates compared to patients admitted to public facilities. Being treated in a public hospital was associated with a higher mortality in the multivariate analysis. In addition, having sepsis diagnosed within one or two hours after the onset of organ dysfunction was significantly associated with reduced mortality in public hospitals but not in private ones.

In our study, the sepsis mortality rate was independently associated with admission to public institutions, although no clear differences could be found between the two types of institutions regarding the severity of illness as assessed by APACHE and SOFA scores. This higher mortality in the public hospitals was already suggested by previous studies [6], [7]. However, in this study, for the first time, public hospitals were independently associated with a higher mortality in a multivariate analysis, where both timely diagnosis and adequate treatment were assessed. There are some possible explanations for our findings.

The time elapsed between the onset of organ dysfunction and the time to the sepsis diagnosis in public hospitals was twice that of private hospitals. This delay in diagnosis might have contributed to our findings of a higher SOFA score, higher delta SOFA and more organ dysfunction in the public hospitals at the moment of diagnosis. These findings reinforce the hypothesis that septic patients in public facilities are recognized late, and ICU admission occurs at a later stage of disease when several organs already presented dysfunction. A previous study in a public emergency room in Brazil demonstrated a significant delay in emergency care and transfer to the ICU, sometimes retarding proper treatment [21]. This observation was corroborated by another study carried out in a Brazilian teaching hospital, where delayed admission of critically ill patients to the ICU (most of them were septic) was associated with increased mortality [22]. There is significant evidence that delayed treatment is associated with a higher mortality; however, there is a paucity of data about the impact of delayed diagnosis, although this conclusion might be considered intuitive. In a public Brazilian hospital, Freitas et al. demonstrated a strict association between the time to sepsis diagnosis and mortality, with a mean delay of 1.7 days [23]. Westphal et al., in a before-after trial in two Brazilian ICUs, showed that the time between the first record in the charts of at least two signs suggestive of infection and the time of diagnosis of severe sepsis was significantly higher in non-survivors. After implementing a sepsis treatment protocol, they were able to show an improvement in sepsis recognition and mortality [24]. Another study also correlated an increased time between the onset of hypoperfusion and the beginning of fluid resuscitation with a higher mortality [25]. All of the findings reported in these studies may be related to deficiencies in the physicians’ knowledge regarding the basic concepts of the disease. Indeed, a previous survey undertaken in Brazil showed that doctors have not mastered the concepts of sepsis and severe sepsis [26]. In this study, we clearly showed that a delay in the sepsis diagnosis was associated with a higher mortality in public institutions. The reason why we could not demonstrate this association in the private hospitals is not clear. Certainly, because the delay in diagnosis is minor in private hospitals, an early recognition would have less impact on the overall quality of care provided. The impact would be even less if the quality of care is better, as it seemed to be in the private institutions where the overall compliance with the bundles was better. It should be noticed that public hospitals had a shorter time to antibiotics and a better compliance with antibiotics and volume/vasopressors. This was expected as in some private hospitals antibiotics could not be delivered in the emergence department before a full admission in the hospital, which needed authorization by the health insurance company. However, it should also be emphasized that as compliance was measured considering the moment of sepsis diagnosis and not the onset of organ dysfunction the delay in sepsis recognition in public settings would potentially create a paradoxical situation of compliance to an important goal not contributing to reduced mortality.

Other potential explanations for the higher mortality rates in the public institutions include an unfavorable patient-healthcare professional ratio, non-optimized processes and a lack of adequate infrastructure in these settings, which could potentially be related to increased mortality. Although we did not assess other quality indicators, indirect evidence of better quality of care and support is our finding of higher daily costs in the private institutions. Moreover, all of the Brazilian studies only analyzed patients admitted to intensive care. This access is more limited in public hospitals, where usually only the more severe cases go to the ICU. Even if properly recognized as sepsis, these patients may arrive at the ICU in a later stage of disease with established organ dysfunction and therefore increased risk of death. Other issues that may contribute to this difference include heterogeneity in health-care access in Brazil [6], mainly in the public health network. Limited access may result in a delay in reaching the emergency services. The time needed to reach emergence services might be quite difference between these two types of institution as well as the percentage of patients transferred from hospital to hospital before a proper diagnosis is made. Differences in global quality of life and health might also play a role, such as worse control of co-morbidities and baseline inadequate nutritional status, baseline socio-economic status, in patients treated in public hospitals.

This study has some strengths. First, this multicenter study evaluated ICUs from several Brazilian hospitals with a considerable number of patients. The inclusion was consecutive during the original study, and the prospective data collection ensures that the variables reported here are robust and reasonably representative of national characteristics. Most of the patients included in the original trial were also evaluated in this one. In addition, the systematic assessment of the time to the sepsis diagnosis is unique in the literature.

This study also has several limitations. First, data from the original study were collected in 2004, and improvements in sepsis recognition and treatment may have occurred since then. However, single center studies and data from the SSC suggest that compliance with the SSC guidelines is still low in Brazil [23], [27], [28]. Second, we were unable to adequately characterize demographic data regarding the ethnicity and socioeconomic status of our population as well as the presence of comorbidities. We also did not collect length of hospital stay or any other long-term mortality, which precludes a cox proportional hazards regression that could have reinforced our findings. Third, we carried out a retrospective analysis of compliance and the time to the sepsis diagnosis. Although the information on compliance could be retrieved from the records more objectively, precisely determining the timing of diagnosis of organ dysfunction and treatments from the charts tend to be more subjective. Another limitation is a probable selection bias because we just analyzed patients admitted to the ICU; thus, our results might not be representative of other hospital settings. It is possible also that our results do not represent the Brazilian reality, as most of our hospitals are located in the Southeast and South regions, the richest regions in Brazil. Actually, it is probable that in the other regions the differences in these two healthcare systems are even greater, as the gap in quality of care between private and public tends to be reinforced in poorest regions. Our hypothesis needs to be confirmed in a prospective study with an adequate sample size and randomly selection of ICU and hospitals in all Brazilian regions.

### Conclusion

We demonstrate that septic patients admitted to Brazilian public hospitals have a higher mortality than those admitted to private hospitals, although they have similar disease severity. Delayed recognition is more frequent in public institutions and this might have been associated with a higher mortality. Thus, improving sepsis recognition and early diagnosis should be important targets to improve the care of septic patients in public institutions.

## Supporting Information

Table S1 **Participating centers with previous inclusion in the original COSTS study and inclusion in the present study.**
(DOCX)

Table S2 **Comparison between the patients included in the present study and those in the missing data group.**
(DOCX)
