# Supplementary material for: Differences in Sepsis Treatment and Outcomes between Public and Private Hospitals in Brazil: A Multicenter Observational Study
Source: PLoS One. 2013 Jun 6;8(6):e64790. doi: 10.1371/journal.pone.0064790 (PMC3675193; doi:10.1371/journal.pone.0064790)
Supplement: Table S1 — Participating centers with previous inclusion in the original COSTS study and inclusion in the present study. (DOCX) [file pone.0064790.s001.docx]

**Supplementary materials**

**Table S1 - Participating centers with previous inclusion in the original COSTS study and inclusion in the present study.**

| **Site** | **City/State** | **Investigador** | **Original number of inclusions** | **Actual number of inclusions** |
| --- | --- | --- | --- | --- |
| Casa Saúde São José | Rio de Janeiro/RJ | André Japiassu | 10 | 7 |
| H. Alípio Correa Neto | São Paulo/ SP | Elcio Tarkieltaub | 21 | 21 |
| H. Base | São José do Rio Preto/SP | Suzana Lobo | 33 | 33 |
| H. Beneficiência Portuguesa | São Paulo/SP | Haggeas Fernandes | 8 | 7 |
| H. das Clínicas | Curitiba/PR | Álvaro Réa Neto | 59 | 55 |
| H. das Clínicas Unesp | Botucatu/SP | Ana Lúcia Gut | 13 | 8 |
| H. Grajaú | São Paulo/ SP | Sérgio Mataloun | 16 | 11 |
| H. Israelita Albert Einstein | São Paulo/ SP | Eliézer Silva | 29 | 29 |
| H. Mater Dei | Belo Horizonte/MG | Anselmo Moura | 38 | 34 |
| H. Municipal São José | Joinvile/SC | Milton Caldeira Filho | 10 | 9 |
| H. Português | Salvador/BA | José Mario Telles | 18 | 14 |
| H. Procardíaco | Rio de Janeiro/RJ | Rubens Costa Filho | 22 | 20 |
| H. Santa Luzia | Brasília/DF | Marcelo Maia | 20 | 20 |
| H. São Lucas | Porto Alegre/RS | Fernando Dias | 23 | 21 |
| H. São Paulo | São Paulo/ SP | Flávia Machado | 58 | 54 |
| H. Sírio Libanês | São Paulo/ SP | Guilherme Schettino | 3 | 2 |
| H. Universitário | Londrina/PR | Cíntia Grion | 48 | 46 |
| H. Vera Cruz | Belo Horizonte/MG | Dinalva Mendes | 6 | 5 |
| Total |  |  | 435 | 396 |
