# Supplementary material for: Differences in Sepsis Treatment and Outcomes between Public and Private Hospitals in Brazil: A Multicenter Observational Study
Source: PLoS One. 2013 Jun 6;8(6):e64790. doi: 10.1371/journal.pone.0064790 (PMC3675193; doi:10.1371/journal.pone.0064790)
Supplement: Table S2 — Comparison between the patients included in the present study and those in the missing data group. (DOCX) [file pone.0064790.s002.docx]

**Table S2. Comparison between the patients included in the present study and those in the missing data group.**

| **Variable** | **Patients in the present study (N=396)** | **Patients with missing data**  **(N=86)** | **p value** |
| --- | --- | --- | --- |
| Age (years) | 62.0 (46.0 - 75.7) | 62.5 (49.5 - 73.0) | 0.75 |
| Male gender | 239 (60.4) | 50 (58.1) | 0.79 |
| APACHE II | 22.0 (16.2 - 28.0) | 22.0 (15.0 - 28.0) | 0.92 |
| SOFA | 7.0 (5.0 - 10.0) | 7.0 (5.0 - 10.0) | 0.37 |
| Type of hospital |  |  | 0.37 |
| Public | 258 (65.2) | 51 (59.3) | - |
| Private | 138 (34.8) | 35 (40.7) | - |
| Organ dysfunctions | 2.0(1.0 - 3.0) | 2.0 (2.0 - 3.0) | 0.95 |
| Cardiovascular dysfunction | 224 (56.6) | 57 (66.3) | 0.09 |
| Respiratory dysfunction | 317 (80.1) | 68 (79.1) | 0.84 |
| Length of ICU stay | 10.0 (5.0 - 18.0) | 11.5 (5.7 - 20.2) | 0.21 |
| ICU mortality | 196 (49.5) | 38 (44.2) | 0.37 |
